# Symptom-Specific Networks and the DBS-Modulated Network in Parkinson’s Disease: A Connectivity-Based Review

**DOI:** 10.3390/brainsci16010016

**Published:** 2025-12-23

**Authors:** Ransheng Huang, Kailiang Wang, Yuqing Zhang, Guoguang Zhao

**Affiliations:** Department of Neurosurgery, Xuanwu Hospital, Capital Medical University, Beijing 100038, China; huangransheng@mail.ccmu.edu.cn (R.H.); klwang@ccmu.edu.cn (K.W.); zhangyuqing@xwh.ccmu.edu.cn (Y.Z.)

**Keywords:** Parkinson’s disease, deep brain stimulation, fMRI, brain network, motor symptom

## Abstract

**Objectives:** With the development of advanced neuroimaging techniques, including resting-state functional magnetic resonance imaging and diffusion tensor imaging, Parkinson’s disease (PD) has increasingly been recognized as a complex brain network disorder. In this review, we summarized research on brain networks in PD to elucidate the network abnormalities underlying its four major motor symptoms and to identify the networks modulated by deep brain stimulation (DBS). **Materials and Methods:** We searched PubMed and Web of Science for the most recent literature on brain network alterations in PD. Eligible studies included those investigating the general PD network (*n* = 10), symptom-specific networks—tremor-dominant (*n* = 13), postural instability and gait disorder (*n* = 9), freezing of gait (*n* = 9), akinetic-rigidity (*n* = 3)—as well as DBS-modulated networks (*n* = 14). Based on these studies, we integrated the findings and used BrainNet Viewer to generate schematic network visualizations. **Results:** The symptom-specific networks exhibited common abnormalities within the sensorimotor network. Evidence from DBS studies suggested that therapeutic effects were associated with modulation of the motor cortex through both functional and structural connectivity. Moreover, the four motor symptoms each demonstrated distinct network features. Specifically, the tremor network was characterized by widespread alterations in the cortico-thalamic-cerebellar circuitry; the postural instability and gait disorder network showed more severe disruptions within the striatum and visual cortex; the freezing of gait network exhibited disruptions in midbrain regions, notably the pedunculopontine nucleus; and the akinetic-rigidity network involved changes in cognition-related networks, particularly the default mode network. **Conclusions:** PD motor symptoms exhibit both distinct network features and shared alterations within the sensorimotor network. DBS modulates large-scale brain networks, especially motor-related networks, contributing to the alleviation of motor symptoms. Characterizing symptom-specific networks may support precision DBS target selection and parameter optimization.

## 1. Introduction

Parkinson’s disease (PD) is a progressive neurodegenerative disorder characterized by impaired motor function with resting tremors, bradykinesia, akinetic-rigidity (AR), postural instability, and gait disturbance. In addition, most patients experience a range of non-motor dysfunctions that are heterogeneous in nature. The stages at which motor symptoms appear vary during the progression of PD [1,2]. Traditionally, PD has been attributed to dopaminergic neuronal degeneration within the substantia nigra (SN) pars compacta and consequent basal ganglia (BG) dysfunction [2,3,4]. However, emerging evidence substantiates a broader conceptualization of PD as a brain network disorder, characterized by aberrant functional and structural connectivity across distributed cortical and subcortical systems. The advent and maturation of sophisticated neuroimaging techniques, particularly resting-state functional magnetic resonance imaging (rs-fMRI) and diffusion tensor imaging (DTI), have enabled in vivo characterization of distributed network abnormalities in PD and facilitated connectome-level investigations [5,6,7,8].

Key implicated networks include the sensorimotor network (SMN), default mode network (DMN), frontoparietal network (FPN), striatal-thalamo-cortical (STC) and cerebello-thalamo-cortical (CTC) circuitry. Dysregulation within and between these networks contributes to distinct clinical phenotypes such as tremor-dominant, postural instability and gait disorder (PIGD), freezing of gait (FoG), and AR subtypes. Network-level analyses afford refined diagnostic and prognostic markers beyond traditional clinical assessments. In particular, deep brain stimulation (DBS), targeting principally the subthalamic nucleus (STN) and globus pallidus internus (GPi), constitutes a cornerstone of advanced PD treatment. Accumulating evidence indicates that DBS-mediated symptomatic improvement arises from modulation of large-scale brain networks, rather than localized effects alone [9]. This review synthesizes contemporary findings on PD-related brain network alterations, delineates symptom-specific network patterns, and characterizes DBS-modulated networks, underscoring the translational potential of symptom-targeted neuromodulation to optimize therapeutic outcomes.

## 2. Materials and Methods

The literature search was conducted using the PubMed and Web of Science databases, covering publications from 2015 to 2025. The search included the following keywords and their combinations: “Parkinson’s disease”, “PD”, “tremor”, “rigidity”, “akinetic” “PIGD”, “FoG”, “functional connectivity”, “structural connectivity”, “deep brain stimulation”, and “brain networks”. This study focused on previously published original research articles related to brain networks in PD. As the aim of the review was to construct symptom-specific functional connectivity networks and DBS modulation networks in PD, the following inclusion criteria were applied: 1) The research explicitly addressed PD and its core motor symptoms (e.g., tremor, rigidity, PIGD, and FG), or clearly examined the network effects of DBS. 2) Studies needed to employ functional or structural connectivity methods to investigate brain network characteristics in PD. 3) Studies further need to report statistically significant region-to-region connections, specifying the anatomical regions involved. To ensure anatomical interpretability and cross-study comparability, only connectivity approaches that permitted explicit mapping between brain regions, such as seed-based functional connectivity and diffusion tractography, were included.

Consequently, studies that did not report anatomically defined connections, such as ICA-only analyses, global graph-theoretical metrics without region-specific edges, or whole-network summaries lacking node-to-node detail, were excluded because these methods do not allow direct construction of symptom-specific anatomical networks.

**Figure 1:**
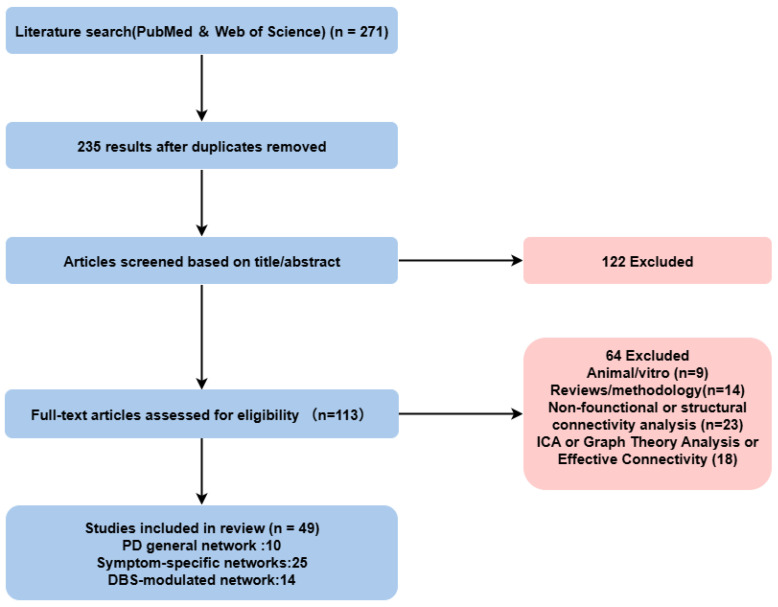
Flowchart depicting the screening process for studies included in this review.

In addition, given the narrative and connectivity-focused nature of this review, we did not apply a formal risk-of-bias assessment tool. However, a qualitative appraisal of included studies was performed, considering participant characterization and PD diagnostic criteria, sample size and subgroup definition, imaging modality and analytical approach, statistical thresholding and correction methods, and the clarity of anatomically defined region-to-region connectivity reporting. Figure 1 outlines our study and search approach.

**Figure 2:**
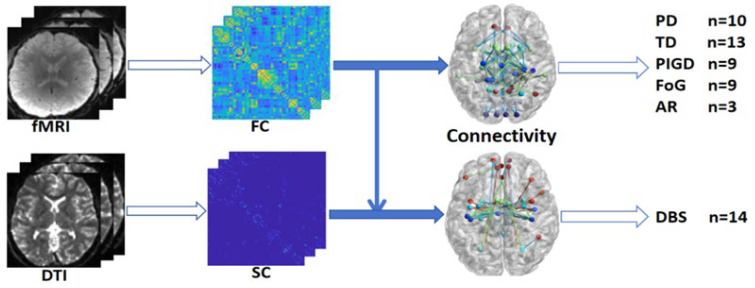
Flowchart illustrating the imaging modalities and connectivity construction methods used in the articles included in this review.

The original article data for constructing both our PD general network and symptom-specific network come from brain region coordinates with significant connectivity changes compared to healthy controls. Most of the included studies used correlation coefficient methods for constructing brain networks by employing rs-fMRI. Regions of interest (ROIs) were defined as seed regions for functional connectivity, after which voxel-wise correlation analysis was conducted. Next Pearson correlation coefficients were calculated the between the average time series within the ROIs and the time series from other brain regions. Research on DBS-modulated networks incorporated rs-fMRI, as well as DTI to construct structural connectivity. White matter fiber tracking or DBS fiber-filtering techniques were used to precisely locate DBS related connections. Imaging modalities employed in the studies included in this review and the visualization approach used for network construction in the present work are provided in Figure 2. The figure also summarizes the number of studies reporting significant findings within the PD general network, tremor network, PIGD network, FOG network, AR network, and DBS-modulated network.

Cortical parcellation was based on Thomas Yeo’s 7-network cortical atlas, which divides the cerebral cortex into seven networks: the DMN, FPN, limbic network, ventral attention network, dorsal attention network, SMN, and visual network [10]. Similarly, cerebellar parcellation was defined according to Buckner’s 7-network cerebellar atlas [11]. In this way, the major resting-state networks of the nodes were determined. Using BrainNet Viewer [12], we visualized the nodes and their connections to provide a clearer representation of the network-level connectivity within the brain.

## 3. General Brain Network Abnormalities in PD

Functional connectivity (FC) in PD patients as revealed by rs-fMRI studies, has provided a comprehensive understanding of general network abnormalities in PD and has been compared with symptom-specific network abnormalities in subsequent sections.

The role of the cerebellum in PD has been highlighted in recent studies. For example, Palmer et al. found widespread alterations in cerebello-cortical FC, changes that were associated with PD-related motor and cognitive impairments [13]. In addition, increased cerebellar local activity and enhanced FC among cerebellar structures have been reported; these findings have been interpreted as potentially reflecting a compensatory mechanism [14,15].

The SMN encompasses the primary sensorimotor, premotor, and supplementary motor area (SMA), and is crucial for voluntary movements [16]. In PD, the SMN exhibits reduced functional connectivity, both internally within the network and between interactions with other networks [17]. Wei et al. found enhanced FC between the SN and sensorimotor regions, suggesting that alterations in the nigro-striatal pathway may be related to motor deficits in PD [18]. Shima et al. focused on striatal dopamine-depleted areas, and found decreased FC with the motor cortex and SN. However, the STN exhibited enhanced FC with the lateral premotor cortex and primary motor cortex (M1) [19].

Cognitive impairment is a critical non-motor symptom of PD. Indeed, with disease progression, the majority of patients eventually develop dementia [20]. Importantly, the DMN is critically involved in cognitive impairment in PD [21,22]. Fathy et al. found reduced FC between the dorsal anterior insula and anterior cingulate cortex in PD. This reduction may lead to destabilization of the DMN and FPN, thereby affecting patients’ cognitive function [23]. Llewelyn et al. discovered that the hippocampus of PD patients showed dysconnectivity with the paracingulate gyri [24]. Chen et al. found that, in the DMN, FC between the posterior cingulate cortex (PCC) and right precuneus, left cuneus, and right angular gyrus was significantly increased [22]. Thibes et al. further showed widespread reduction in FC between the PCC and motor cortex [25]. Wang and colleagues determined that enhanced FC between the ventromedial putamen and left paracentral lobule, as well as multiple nodes of the DMN in PD patients [26].

**Figure 3:**
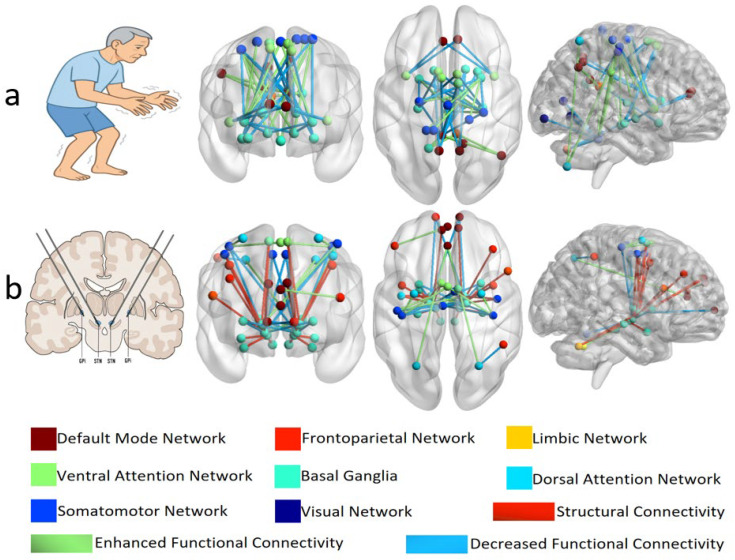
The PD general network (**a**) exhibits extensive FC alterations between the basal ganglia, SMN, and DMN. The DBS-modulated network (**b**) shows that DBS stimulation exerts significant effects on the motor cortex. Nodes represent brain regions (color-coded by functional network), and edges represent connectivity changes (green: enhanced FC; blue: decreased FC; red: structural connectivity). Edges are undirected and line thickness does not encode effect size (used for visualization only). Visualization is based on PD general network studies (*n* = 10) in (**a**) and DBS-modulated network studies (*n* = 14) in (**b**). Abbreviations: SMN, sensorimotor network; DMN, default mode network.

The PD general network exhibits extensive FC alterations between the basal ganglia, SMN, and DMN. These findings underscore that PD reflects widespread large-scale network disruptions rather than pathology restricted to the SN. A total of 10 studies were included in this research, and the relevant results are summarized and visualized in Figure 3a.

## 4. Symptom-Specific Brain Network Abnormalities in PD

### 4.1. The Tremor Network

Tremor is typically characterized as an involuntary, rhythmic shaking that primarily occurs at rest and is therefore referred to as resting tremor. It is often one of the earliest symptoms observed in patients with PD. The tremor-dominant subtype of PD (PD-TD) is distinguished by resting tremor as its primary feature [27]. Compared to other subtypes, PD-TD progresses more slowly, poses a lower risk of cognitive impairment, and responds more favorably to DBS therapy [28].

Helmich et al. proposed a model in which, in PD-TD, the basal ganglia trigger tremor episodes, whereas the CTC circuit is associated with producing the tremor [29]. The cerebellum and the STN are crucial hubs of these two loops [30,31,32]. In PD-TD, functional coupling within and between these hubs is markedly altered. Multiple studies have demonstrated abnormal bidirectional cerebello-cortical coupling in PD-TD, particularly between the cerebellum and motor cortex [33,34,35]. In addition, cerebello-prefrontal FC has also been reported to be disrupted, indicating that tremor-related cerebellar dysfunction extends beyond the motor loop [36,37]. Similarly, FC between the STN and the motor cortex, as well as between the STN and the cerebellum, is disturbed in tremor patients [34,38,39]. Another key node shared by these two circuits is the primary motor cortex. In PD-TD, M1 shows not only abnormal FC with the basal ganglia and the cerebellum [40], but also increased connectivity with the pedunculopontine nucleus (PPN) and the inferior parietal lobule [41]. These convergent alterations at the main nodes of the basal ganglia and the CTC circuit support the view that network-level dysfunction within these loops plays a central role in the generation and modulation of Parkinsonian tremor.

In addition, PD patients have been found to increasingly rely on visual input to control their movements. The occipital lobe is an important component of the visual network [42], and studies in PD-TD have reported reduced functional connectivity between the occipital gyrus and other cortical regions [43] suggesting that visual network alterations may accompany tremor network reorganization. However, this feature is more pronounced in PIGD patients.

**Figure 4:**
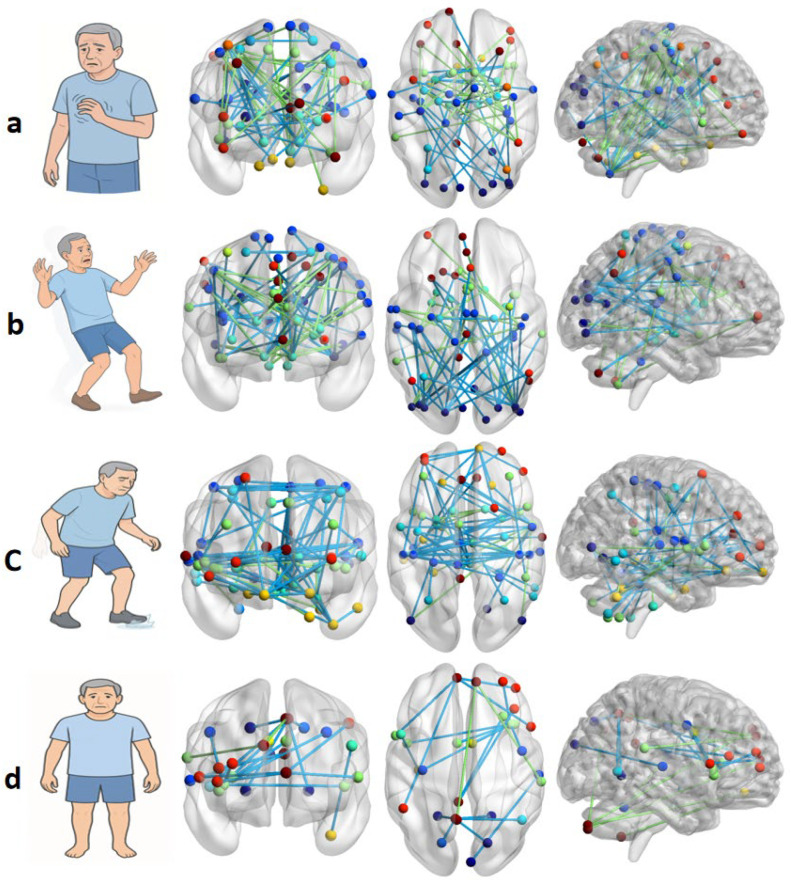
Symptom-specific brain networks in Parkinson’s disease. (**a**) Tremor-dominant network (*n* = 13); (**b**) postural instability and gait disorder (PIGD) network (*n* = 9); (**c**) freezing of gait (FoG) network (*n* = 9); and (**d**) akinetic-rigidity (AR) network (*n* = 3). Nodes represent anatomically defined brain regions, and edges indicate statistically significant connectivity changes in each PD subtype compared with healthy controls (HC). Blue edges denote decreased FC and green edges denote increased FC (relative to HC). Color coding and network definitions are consistent with those shown in Figure 3. Edges are undirected and line thickness does not encode effect size (used for visualization only).

The tremor network is characterized by FC alterations in the CTC circuit and basal ganglia. In particular, the FC among the cerebellum, STN, and motor cortex shows notably prominent alterations. It is noteworthy that the DMN shows relatively limited FC alterations within the tremor network. This finding may be associated with the lower risk of cognitive impairment in the PD-TD subtype. Overall, these findings are consistent with abnormal oscillatory interactions within coupled the CTC and basal ganglia circuits. In total, 13 studies were included in the analysis of the tremor network. These observations are collectively summarized and illustrated in Figure 4a.

### 4.2. The PIGD Network

The PIGD subtype (PD-PIGD) is characterized primarily by impaired postural control and gait disturbances, including slow walking, difficulty in initiating movement, and balance instability [27]. It is more commonly observed in the later stages of the disease and has a direct impact on patients’ daily functioning and quality of life. Compared to PD-TD, PD-PIGD is often associated with more rapid disease progression, more severe cognitive impairment, and poorer responsiveness to treatment [44,45].

Across resting-state fMRI studies, PD-PIGD shows predominantly reduced FC within the STC circuitry, including decreased intrinsic coupling between the caudate and putamen and weakened connectivity between the STN and putamen and pons [39,41]. In addition, PD-PIGD exhibits reduced FC across multiple cortical regions, including the dorsolateral superior frontal gyrus, SMA, and M1, with the occipital cortex showing particularly widespread decreases in connectivity [40,43,46].

Both PD-TD and PD-PIGD share several compensatory mechanisms, including increased cerebellum–SMN FC and increased STN connectivity with the sensorimotor cortex and visual cortex [35,38,47]. However, hyperconnectivity between the PPN and the sensorimotor cortex has been reported exclusively in PD-PIGD [41].

A total of nine studies were included in the analysis of the PIGD network. The summarized findings suggest that the PIGD and TD networks share some common patterns of injury and compensatory mechanisms. However, the PIGD subtype appears to exhibit more severe FC disruptions within the striatum and visual cortex, potentially reflecting impaired visuomotor integration underlying balance deficits and fall risk in PD-PIGD patients. A schematic summary of these findings is illustrated in Figure 4b.

### 4.3. The FoG Network

FoG is characterized by brief episodes of an inability to step or by extremely short steps. These episodes typically occur at gait initiation or when turning during walking [48]. Although FoG is considered a specific motor manifestation within the PIGD subtype, it is closely associated with disease progression and non-motor symptoms in all PD patients [49]. Unlike other symptoms of PD, FoG shows limited responsiveness to conventional medical and surgical treatments [50].

Both structural damage to the midbrain and a marked reduction in midbrain–cortical connectivity have been established in PD-FoG patients [51]. The PPN, a major nucleus of the mesencephalic locomotor region, plays a crucial role in FoG pathogenesis, and PD-FoG patients exhibit abnormal PPN FC with corticopontine–cerebellar pathways [52,53]. The dorsal raphe nucleus, the principal serotonergic nucleus in the brainstem, also shows significantly reduced FC with the SMA, left superior frontal gyrus and left median cingulate cortex [54,55].

In PD-FoG patients, widespread reduced FC has been observed within the SMN, DMN, and visual network [56]. These include impaired FC between the left parietal opercular cortex and primary somatosensory and auditory areas [57], between the right middle frontal gyrus and DMN nodes [58] and between the right lateral posterior thalamic nuclei and the right inferior parietal lobule [59], as well as disrupted connectivity within frontoparietal–limbic–striatal circuits and decreased cerebellar–prefrontal FC [60,61].

A total of nine studies were included in the analysis of the FoG network. Compared to other networks, this network appears to exhibit more pronounced structural and functional alterations in the midbrain, indicating that midbrain structures, notably the PPN, likely play a pivotal role in FoG pathophysiology. These abnormalities are proposed to disrupt the interaction between the mesencephalic locomotor region and cortical executive and SMN, leading to impaired automatic gait control and increased reliance on ineffective cortical compensation during gait initiation and turning [51,62]. In addition, widespread FC alterations are also observed in the SMN, DMN, visual network, and cerebellum. The involved brain structures within the FoG network are illustrated in Figure 4c.

### 4.4. The AR Network

PD-AR is primarily characterized by akinesia, bradykinesia, and rigidity. PD-AR patients are more prone to developing depressive symptoms and cognitive decline than other subtypes [63]. Previous studies have also indicated that these patients are particularly vulnerable to reductions in procedural learning and memory [64]. Depression and cognitive impairment also significantly impact the quality of life in PD-AR patients.

The right fronto-insular cortex (rFIC) is a critical node of cognitive control shows reduced FC with the salience network [65,66]. The DMN is critically involved in cognitive processes [67]. In PD-AR, a decline in FC within the posterior DMN, accompanied by enhanced compensatory FC in the anterior DMN [68]. Hu et al. further reported distinct FC patterns between the PD-TD and PD-AR subtypes. Specifically, PD-TD patients engaged greater cerebellar resources, whereas those with the AR subtype predominantly recruited frontal lobe resources [69].

A total of three studies investigating the AR network were included. Summarizing the findings, limited evidence suggests that PD-AR patients may show alterations in cognition-related networks, including the DMN, which is implicated in multiple cognitive processes. However, because only three studies were included, these observations should be interpreted cautiously and warrant further validation. These findings are consistent with previous studies indicating that PD-AR patients are more prone to cognitive decline. The summarized results are illustrated in Figure 4d. In addition, the key abnormalities across the four symptom-specific networks are summarized in Table 1.

### 4.5. DBS-Modulated Network

DBS is a well-established treatment for PD, of which the STN and GPi are the two major targets [70]. DBS modulated multi-network abnormalities and the resulting FC changes have been shown to be associated with improvements in motor and psychiatric symptoms in PD patients [71]. Different network nodes may be modulated by different stimulation targets, potentially resulting in similar clinical outcomes [72]. Therefore, we summarized the structural and functional connectivity features of the volume of tissue activated (VTA) in both STN-DBS and GPi-DBS from previous studies to identify the modulated network of DBS in PD.

STN-DBS may exert its effects not only by directly stimulating the STN but also by modulating distant brain regions via white matter fiber pathways [73]. Studies have shown that the STN-VTA connects to the brainstem, cerebellum, and motor and premotor cortices [9,72,74,75,76,77]. The hyperdirect pathway (HDP), which connects the STN and motor cortex, has been confirmed in multiple studies and may represent a potential therapeutic target in PD [9,74]. Indeed, Chen et al. revealed that STN-DBS may deactivate the motor cortex [78].

Furthermore, Accolla et al. suggested that the STN is also connected with limbic and associative areas, as well as medial temporal regions [75]. Calvano et al. further showed connections of the STN with the prefrontal cortex [77]. Bai et al. demonstrated that DBS modulates FC within the SMN and across multiple networks centered on the FPN. The authors further indicated that such effects are symptom-specific [71]. Shen et al. identified two distinct circuits: the GPi circuit that selectively responded to high-frequency stimulation and was associated with overall motor improvement; and the M1 circuit that exhibited progressive deactivation over time and was linked to reduced bradykinesia [79].

DBS may alleviate specific motor symptoms through modulation of distinct white matter tracts. For example, tremor improvement has been associated with stimulation of pathways connected to the M1 and cerebellum, whereas axial symptom improvement has been associated with pathways connected to the SMA and brainstem. Bradykinesia and rigidity improvements have been associated with pathways connected to the supplementary motor and premotor cortices, respectively [80]. In addition, improvement in FoG has been linked to stimulation volumes structurally connected to motor areas, the prefrontal cortex, and the GPi, whereas stimulation of the lenticular fasciculus may be associated with worsening of FoG [81].

Moreover, the functional activity of the motor cortex induced by DBS varies across different motor phenotypes. DiMarzio et al. observed that DBS led to deactivation of the M1 in TD-PD patients but activation of both the M1 and SMA in the PD-AR subtype. In addition, STN-DBS resulted in M1 activation in PD-PIGD patients, whereas GPi-DBS resulted in M1 deactivation [82].

In the classical model of the BG, under Parkinsonian conditions, the loss of dopamine in the SN leads to hypoactivity in the direct pathway and hyperactivity in the indirect pathway. These alterations result in reduced inhibition from the striatum to the GPi and increased excitation from the STN, ultimately causing excessive output from the GPi, leading to excessive inhibition of the thalamus and cortex, which results in motor suppression [32].

Multiple studies have demonstrated that both STN-DBS and GPi-DBS improve motor symptoms and functioning in PD patients. Although STN-DBS has been reported to produce greater motor improvements, no significant differences between the two targets have been observed in cognition, mood, or behavior [83,84]. These overlapping clinical effects are consistent with the fact that both STN-DBS and GPi-DBS engage motor-related CTC and basal ganglia circuitry, showing partially overlapping modulation of SMN. However, STN-DBS tends to exert stronger effects on SMA connectivity and more consistently modulates connections with cerebellum pathways and motor thalamus, whereas GPi-DBS shows relatively stronger links with primary motor and sensory cortex [85,86].

The DBS-modulated network included 14 articles. Overall, DBS stimulation was found to exert significant effects on the motor cortex, particularly the M1 and SMA, which likely underlie its therapeutic efficacy. Furthermore, compared with GPi-DBS, STN-DBS affected a broader range of brain structures and exerted stronger modulatory effects on the motor cortex. Importantly, the modulatory effects of DBS also differ significantly across distinct motor symptoms. We have illustrated the modulate network of DBS in Figure 3b.

## 5. Discussion

With advances in research on brain network disorders and the development of advanced imaging techniques, such as fMRI and DTI, it has become possible to trace and analyze abnormal signal transmission within brain networks [87]. PD is not merely the loss of dopaminergic neurons in the SN, but it also involves dysregulation across multiple brain regions. Overall, these findings provide deeper insight into PD-related network disturbances and the phenotype-specific effects of DBS.

The construction of PD general brain network abnormalities is based on fMRI studies comparing PD patients with healthy controls. For this reason, the PD general network encompasses broader alterations in resting-state networks and brain structures compared to symptom-specific brain network abnormalities. For instance, network-level and cross-network alterations involving the SMN, DMN, FPN, cerebellum, brainstem, and BG have been consistently demonstrated. Dysfunction within the CTC circuit is thought to be strongly associated with the core motor manifestations of PD, while the DMN predominantly contributes to non-motor symptoms. FC disruptions have been identified both within the cerebellum and between the cerebellum and cortical regions, and such cerebello–cortical alterations have been associated with impairments in motor coordination, balance control, and also cognitive performance in PD [13,14]. Consistent with this, increased cerebellar FC in patients with frequent falls has been interpreted as reflecting a compensatory recruitment additional network resources to counteract gait and postural instability [14]. At the large-scale network level, altered FC within the DMN and between DMN hubs, such as the precuneus and posterior cingulate cortex, and motor systems has been associated with cognitive impairment and with reduced efficiency of cognitive–motor integration in PD [22,23,25].

In the context of PD symptom-specific brain network abnormalities, each symptom is characterized by distinctive functional network alterations, yet overlapping patterns can also be observed across symptoms.

The tremor network has received the most extensive investigation. Connectivity changes centered on the cerebellum, BG, and motor cortex are particularly prominent in the tremor subtype, which are consistently interpreted as reflecting abnormal rhythm generation and amplification within coupled CTC and basal ganglia–thalamo–cortical circuits [33,36,38,39]. In particular, abnormal coupling between the dentate nucleus and the primary motor cortex has been directly associated with tremor severity, supporting a central role of cerebellar output in tremor maintenance [36]. Consistent with this oscillatory network architecture, DBS targeting the CTC circuitry appears to be more effective compared with stimulation of other networks [28].

The PIGD network shares certain similarities with the tremor network; however, PD-PIGD patients exhibit more pronounced FC impairments within the visual cortex and visuospatial associative regions [37,43]. These alterations are likely associated with the increased risk of falls and balance deficits observed in these patients, as impaired visual–motor integration and reduced efficiency of using visuospatial cues can weaken postural control during gait and turning [41,58]. The emergence of FoG indicates that PD has progressed to an advanced stage, where both pharmacological therapy and DBS show limited efficacy. Widespread reductions in functional connectivity have been observed across the whole brain within the FoG networks, including midbrain, frontal, parietal, and cerebellar regions [53,57]. Notably, alterations in midbrain connectivity seem to play a critical role [52,63], and have been proposed to disrupt interactions between brainstem locomotor circuits and frontal executive network and SMN, leading to loss of automatic gait control and increased reliance on vulnerable executive compensation during gait initiation and turning [51,61].

PD-AR is frequently accompanied by depression and cognitive impairment, both of which significantly compromise patients’ quality of life. Within the AR network, the DMN, which is closely linked to cognitive processing, exhibits marked FC disruptions [68], suggesting early vulnerability of large-scale cognitive networks in rigidity-dominant PD and providing a network basis for depressive and cognitive symptoms. The SMN encompasses the somatosensory and motor cortices and extends into the SMA, playing a crucial role in the initiation of voluntary movements. In AR patients, reduced connectivity and altered fronto-insular coupling have been shown to impair the switching between executive and motor networks [66,69], thereby elevating the threshold for voluntary movement initiation and contributing to bradykinesia and rigidity.

Alterations in the SMN are consistently observed across all four symptom-specific networks and are also prominent in the PD general network, underscoring its critical role. Correcting or mitigating these abnormalities may represent an important network-level mechanism contributing to motor symptom improvement in PD.

DBS delivers electrical pulses to specific targets, such as the STN and GPi. Through connected white matter tracts and neural circuits, particularly the HDP linking the STN with the (pre)motor cortex, DBS may modulate large-scale distributed networks to achieve therapeutic effects [9]. A sophisticated understanding of how the brain responds to DBS is a prerequisite for enhancing its therapeutic efficacy and minimizing potential side effects [79]. A synthesis of previous research indicates that the effects of DBS are not limited to motor-related regions such as the SMN and cerebellum, but also extend to networks associated with non-motor symptoms. Although this review focuses on motor networks, non-motor circuits, including limbic and associative networks, may also be engaged by DBS-related modulation and warrant further investigation. While DBS can provide substantial motor improvements, optimizing each motor symptom may require stimulation of distinct white matter tracts. If electrodes are not ideally placed or if postoperative programming of DBS parameters, which still largely relies on subjective experience, is suboptimal, motor symptom control may be inadequate, and side effects may occur [80,88]. DBS is the most effective surgical treatment for PD, with its efficacy validated over the past three decades. A thorough understanding of symptom-specific network abnormalities in PD and the neural mechanisms underlying DBS treatment effects is essential for optimizing therapeutic outcomes and guiding clinicians in surgical planning and postoperative programming. Emerging DBS technologies, such as directional and adaptive DBS, may offer hardware-based solutions, yet targeting symptom-specific networks and understanding the symptom-specific DBS modulation remain pivotal for advancing PD DBS therapy.

## 6. Limitations

An important methodological limitation of the current literature is that symptom-specific networks in Parkinson’s disease are predominantly inferred from resting-state fMRI, which captures correlational relationships rather than causal interactions. Furthermore, because many network studies rely on ICA or graph-theoretical metrics, our edge-based synthesis (requiring anatomically defined node-to-node connections) may under-represent data-driven whole-brain network components and topological reorganization, thereby affecting the completeness of symptom-specific network mapping. By contrast, DBS-modulated networks are commonly derived by combining diffusion-based tractography with functional imaging, where DTI delineates structural constraints between brain regions and fMRI reflects stimulation-associated network patterns. While these approaches provide valuable anatomical and network-level insights, they do not permit direct inference of causal or dynamic network interactions.

Regarding DBS connectomics, findings may vary across studies due to methodological differences in electrode localization and VTA modeling assumptions, which can limit comparability across studies. Additional heterogeneity may arise from DBS target (STN vs GPi), medication state, and stimulation parameters; although our included DBS studies predominantly reflect chronic postoperative imaging, variability in study protocols may still affect reported connectivity patterns. In addition, tract and network inferences can be influenced by atlas or parcellation choices, which may affect the definition of nodes and pathways across studies. Finally, patient heterogeneity (e.g., disease stage, symptom profile, and individual anatomy or connectivity) may confound group-level inferences. Consequently, associations between network alterations and symptom improvement should be interpreted with caution.

## 7. Future Implications

DBS remains the most effective surgical treatment for advanced stages of PD. Although it may be accompanied by side effects, its efficacy has been validated through decades of clinical practice and research. With the growing understanding of PD, accumulating evidence from brain network studies suggests that motor improvements are closely linked to the modulation of symptom-specific network abnormalities.

Thus, tailoring DBS strategies to individual patients, by selecting the most appropriate targets and optimizing stimulation parameters to precisely modulate symptom-specific networks, represents a promising direction for enhancing therapeutic efficacy while minimizing adverse effects. In recent years, technological advances such as directional DBS and adaptive DBS have enabled more precise modulation of one or multiple symptom-specific networks. However, this also imposes a substantial burden on clinicians in terms of surgical planning and postoperative programming. The investigation of symptom-specific DBS-modulated networks is therefore essential to future progress. Importantly, these efforts should not be limited to the classical STN and GPi targets; novel targets, such as the pedunculopontine nucleus and cerebellar regions, also warrant further exploration. Yet, current research in these areas remains limited. Emerging evidence consistently implicates cerebellar circuits in tremor network and the PPN within locomotor network relevant to PIGD and FoG. However, both targets still lack large-scale, multicenter, prospective studies to rigorously validate their clinical efficacy and safety. Moreover, recent work suggests that optimal improvement of different motor symptoms may correspond to distinct white-matter connectivity patterns [80]. Nevertheless, how stimulation of these symptom-relevant pathways translates into symptom-specific functional network dynamics remains insufficiently characterized.

The convergence of innovative DBS technologies with advancing knowledge of symptom-specific brain networks is expected to drive significant breakthroughs in PD therapy, while simultaneously presenting new challenges for clinical and research scientist.

## 8. Conclusions

In this review, we discussed the symptom-specific brain network abnormalities in PD and the DBS-modulated network. The major symptoms of PD not only exhibit distinct network features but also share common alterations in the functional connectivity of the SMN. By stimulating specific targets, DBS modulates large-scale brain networks, particularly the SMN, thereby alleviating motor symptoms of PD. Furthermore, the differential responses of various symptoms to DBS underscore the need for further investigation into symptom-specific DBS-modulated networks.

## Figures and Tables

**Table 1:** Key symptom-specific functional network abnormalities in Parkinson’s disease.

Symptom Domain	Key Nodes/Circuits	Functional Connectivity Abnormalities (rs-fMRI)
**Tremor (PD-TD)**	Cerebello-thalamo-cortical circuit; basalganglia-motor cortex	Altered cerebello-motor and STN-motor coupling; relative sparing of DMN
**Postural Instability and gait disorder (PD-PIGD)**	Striato-thalamo-cortical circuitry; visual networks	Reduced striatal and cortical motor FC pronounced visual-motor FC impairment
**Freezing of gait (PD-FoG)**	Midbrain-cortical circuitry; frontal-parietal networks	Widespread FC reduction with prominent midbrain-cortical disruption
**Akinetic-rigidity (PD-AR)**	Default mode network (DMN); executive-motor networks	Disrupted DMN connectivity and impaired executive-motor network coupling

This table provides a side-by-side comparison of key symptom-specific functional network abnormalities in Parkinson’s disease, based on resting-state fMRI findings summarized in the main text.

## Data Availability

No new data were created or analyzed in this study. Data sharing is not applicable to this article.

## Abbreviations

The following abbreviations are used in this manuscript:

PD	Parkinson’s disease
DBS	Deep brain stimulation
AR	Akinetic-rigidity
SN	Substantia nigra
BG	Basal ganglia
STC	Striatal–thalamo–cortical circuitry
CTC	Cerebello–thalamo–cortical circuitry
rs-fMRI	Resting-state functional magnetic resonance imaging
DTI	Diffusion tensor imaging
SMN	Sensorimotor network
DMN	Default mode network
FPN	Frontoparietal network
PIGD	Postural instability and gait disorder
FoG	Freezing of gait
STN	Subthalamic nucleus
GPi	Globus pallidus internus
ROIs	Regions of interest
FC	Functional connectivity
SMA	Supplementary motor area
PCC	Posterior cingulate cortex
PD-TD	The tremor-dominant subtype of PD
PPN	Pedunculopontine nucleus
PD-PIGD	The PIGD subtype of PD
PD-FoG	The FoG subtype of PD
PD-AR	The AR subtype of PD
VTA	Volume of tissue activated
HDP	Hyperdirect pathway
rFIC	Right fronto-insular cortex

## References

[1] Marras C., Lang A. (2013). Parkinson’s disease subtypes: Lost in translation?. J. Neurol. Neurosurg. Psychiatry.

[2] Kalia L.V., Lang A.E. (2015). Parkinson’s disease. Lancet Lond. Engl..

[3] Sveinbjornsdottir S. (2016). The clinical symptoms of Parkinson’s disease. J. Neurochem..

[4] Bartels A.L., Leenders K.L. (2009). Parkinson’s disease: The syndrome, the pathogenesis and pathophysiology. Cortex J. Devoted Study Nerv. Syst. Behav..

[5] Ellis E.G., Meyer G.M., Kaasinen V., Corp D.T., Pavese N., Reich M.M., Joutsa J. (2024). Multimodal neuroimaging to characterize symptom-specific networks in movement disorders. NPJ Park. Dis..

[6] Prodoehl J., Burciu R.G., Vaillancourt D.E. (2014). Resting state functional magnetic resonance imaging in Parkinson’s disease. Curr. Neurol. Neurosci. Rep..

[7] Bae Y.J., Kim J.-M., Sohn C.-H., Choi J.-H., Choi B.S., Song Y.S., Nam Y., Cho S.J., Jeon B., Kim J.H. (2021). Imaging the Substantia Nigra in Parkinson Disease and Other Parkinsonian Syndromes. Radiology.

[8] Alexander A.L., Lee J.E., Lazar M., Field A.S. (2007). Diffusion tensor imaging of the brain. Neurother. J. Am. Soc. Exp. Neurother..

[9] Vassal F., Dilly D., Boutet C., Bertholon F., Charier D., Pommier B. (2020). White matter tracts involved by deep brain stimulation of the subthalamic nucleus in Parkinson’s disease: A connectivity study based on preoperative diffusion tensor imaging tractography. Br. J. Neurosurg..

[10] Thomas Yeo B.T., Krienen F.M., Sepulcre J., Sabuncu M.R., Lashkari D., Hollinshead M., Roffman J.L., Smoller J.W., Zöllei L., Polimeni J.R. (2011). The organization of the human cerebral cortex estimated by intrinsic functional connectivity. J. Neurophysiol..

[11] Buckner R.L., Krienen F.M., Castellanos A., Diaz J.C., Yeo B.T.T. (2011). The organization of the human cerebellum estimated by intrinsic functional connectivity. J. Neurophysiol..

[12] Xia M., Wang J., He Y. (2013). BrainNet Viewer: A network visualization tool for human brain connectomics. PLoS ONE.

[13] Palmer W.C., Cholerton B.A., Zabetian C.P., Montine T.J., Grabowski T.J., Rane S. (2021). Resting-State Cerebello-Cortical Dysfunction in Parkinson’s Disease. Front. Neurol..

[14] Kaut O., Mielacher C., Hurlemann R., Wüllner U. (2020). Resting-state fMRI reveals increased functional connectivity in the cerebellum but decreased functional connectivity of the caudate nucleus in Parkinson’s disease. Neurol. Res..

[15] Xu S., He X.-W., Zhao R., Chen W., Qin Z., Zhang J., Ban S., Li G.-F., Shi Y.-H., Hu Y. (2019). Cerebellar functional abnormalities in early stage drug-naïve and medicated Parkinson’s disease. J. Neurol..

[16] Wu T., Long X., Wang L., Hallett M., Zang Y., Li K., Chan P. (2011). Functional connectivity of cortical motor areas in the resting state in Parkinson’s disease. Hum. Brain Mapp..

[17] Caspers J., Rubbert C., Eickhoff S.B., Hoffstaedter F., Südmeyer M., Hartmann C.J., Sigl B., Teichert N., Aissa J., Turowski B. (2021). Within- and across-network alterations of the sensorimotor network in Parkinson’s disease. Neuroradiology.

[18] Wei L., Hu X., Yuan Y., Liu W., Chen H. (2018). Abnormal ventral tegmental area-anterior cingulate cortex connectivity in Parkinson’s disease with depression. Behav. Brain Res..

[19] Shima A., Inano R., Tabu H., Okada T., Nakamoto Y., Takahashi R., Sawamoto N. (2023). Altered functional connectivity associated with striatal dopamine depletion in Parkinson’s disease. Cereb. Cortex Commun..

[20] Aarsland D., Andersen K., Larsen J.P., Lolk A., Kragh-Sørensen P. (2003). Prevalence and characteristics of dementia in Parkinson disease: An 8-year prospective study. Arch. Neurol..

[21] Olde Dubbelink K.T.E., Schoonheim M.M., Deijen J.B., Twisk J.W.R., Barkhof F., Berendse H.W. (2014). Functional connectivity and cognitive decline over 3 years in Parkinson disease. Neurology.

[22] Chen L., Huang T., Ma D., Chen Y.-C. (2022). Altered Default Mode Network Functional Connectivity in Parkinson’s Disease: A Resting-State Functional Magnetic Resonance Imaging Study. Front. Neurosci..

[23] Fathy Y.Y., Hepp D.H., De Jong F.J., Geurts J.J.G., Foncke E.M.J., Berendse H.W., Van De Berg W.D.J., Schoonheim M.M. (2020). Anterior insular network disconnection and cognitive impairment in Parkinson’s disease. NeuroImage Clin..

[24] Llewelyn L.E., Kornisch M., Park H., Ikuta T. (2022). Hippocampal Functional Connectivity in Parkinson’s Disease. Neurodegener. Dis..

[25] Thibes R.B., Novaes N.P., Lucato L.T., Campanholo K.R., Melo L.M., Leite C.C., Amaro E., Barbosa E.R., Bor-Seng-Shu E., Cardoso E.F. (2017). Altered Functional Connectivity Between Precuneus and Motor Systems in Parkinson’s Disease Patients. Brain Connect..

[26] Wang H., Xu J., Yu M., Ma X., Li Y., Pan C., Ren J., Liu W. (2022). Altered Functional Connectivity of Ventral Striatum Subregions in De-novo Parkinson’s Disease with Depression. Neuroscience.

[27] Jankovic J., McDermott M., Carter J., Gauthier S., Goetz C., Golbe L., Huber S., Koller W., Olanow C., Shoulson I. (1990). Variable expression of Parkinson’s disease: A base-line analysis of the DATATOP cohort. The Parkinson Study Group. Neurology.

[28] Eggers C., Pedrosa D.J., Kahraman D., Maier F., Lewis C.J., Fink G.R., Schmidt M., Timmermann L. (2012). Parkinson subtypes progress differently in clinical course and imaging pattern. PLoS ONE.

[29] Helmich R.C., Hallett M., Deuschl G., Toni I., Bloem B.R. (2012). Cerebral causes and consequences of parkinsonian resting tremor: A tale of two circuits?. Brain J. Neurol..

[30] Helmich R.C., Janssen M.J.R., Oyen W.J.G., Bloem B.R., Toni I. (2011). Pallidal dysfunction drives a cerebellothalamic circuit into Parkinson tremor. Ann. Neurol..

[31] Wu T., Hallett M. (2013). The cerebellum in Parkinson’s disease. Brain J. Neurol..

[32] DeLong M.R. (1990). Primate models of movement disorders of basal ganglia origin. Trends Neurosci..

[33] Prasad S., Saini J., Bharath R.D., Pal P.K. (2024). Differential patterns of functional connectivity in tremor dominant Parkinson’s disease and essential tremor plus. J. Neural Transm..

[34] Shen B., Yao Q., Li W., Dong S., Zhang H., Zhao Y., Pan Y., Jiang X., Li D., Chen Y. (2024). Dynamic cerebellar and sensorimotor network compensation in tremor-dominated Parkinson’s disease. Neurobiol. Dis..

[35] Chen Z., He C., Zhang P., Cai X., Huang W., Chen X., Xu M., Wang L., Zhang Y. (2023). Abnormal cerebellum connectivity patterns related to motor subtypes of Parkinson’s disease. J. Neural Transm..

[36] Ma H., Chen H., Fang J., Gao L., Ma L., Wu T., Hou Y., Zhang J., Feng T. (2015). Resting-state functional connectivity of dentate nucleus is associated with tremor in Parkinson’s disease. J. Neurol..

[37] Yin L., Zhu Z., Fu J., Zhou C., Liu Z., Li Y., Luo Z., Zhu Y., Xu Z., Yang X. (2024). Differences in gray matter atrophy and functional connectivity between motor subtypes of Parkinson’s disease. Acta Neurol. Belg..

[38] Wang Z., Chen H., Ma H., Ma L., Wu T., Feng T. (2016). Resting-state functional connectivity of subthalamic nucleus in different Parkinson’s disease phenotypes. J. Neurol. Sci..

[39] Hou Y., Ou R., Yang J., Song W., Gong Q., Shang H. (2018). Patterns of striatal and cerebellar functional connectivity in early-stage drug-naïve patients with Parkinson’s disease subtypes. Neuroradiology.

[40] Wang Q., Yu M., Yan L., Xu J., Wang Y., Zhou G., Liu W. (2023). Altered functional connectivity of the primary motor cortex in tremor dominant and postural instability gait difficulty subtypes of early drug-naive Parkinson’s disease patients. Front. Neurol..

[41] Vervoort G., Alaerts K., Bengevoord A., Nackaerts E., Heremans E., Vandenberghe W., Nieuwboer A. (2016). Functional connectivity alterations in the motor and fronto-parietal network relate to behavioral heterogeneity in Parkinson’s disease. Parkinsonism Relat. Disord..

[42] Kravitz D.J., Saleem K.S., Baker C.I., Mishkin M. (2011). A new neural framework for visuospatial processing. Nat. Rev. Neurosci..

[43] Lan Y., Yuan H., Ma X., Yin C., Liu X., Zeng X., Lyu J., Xiong Y., Zhang X., Lu H. (2024). Resting-state functional connectivity of the occipital cortex in different subtypes of Parkinson’s disease. CNS Neurosci. Ther..

[44] Montgomery E.B. (2006). Practice parameter: Diagnosis and prognosis of new onset Parkinson disease (an evidence-based review): Report of the Quality Standards Subcommittee of the American Academy of Neurology. Neurology.

[45] Ba F., Obaid M., Wieler M., Camicioli R., Martin W.R.W. (2016). Parkinson Disease: The Relationship Between Non-motor Symptoms and Motor Phenotype. Can. J. Neurol. Sci. J. Can. Sci. Neurol..

[46] Xing F. (2024). Altered connectivity between frontal cortex and supplementary motor area in various types of Parkinson’s disease. Am. J. Transl. Res..

[47] Shen B., Gao Y., Zhang W., Lu L., Zhu J., Pan Y., Lan W., Xiao C., Zhang L. (2017). Resting State fMRI Reveals Increased Subthalamic Nucleus and Sensorimotor Cortex Connectivity in Patients with Parkinson’s Disease under Medication. Front. Aging Neurosci..

[48] Nutt J.G., Bloem B.R., Giladi N., Hallett M., Horak F.B., Nieuwboer A. (2011). Freezing of gait: Moving forward on a mysterious clinical phenomenon. Lancet Neurol..

[49] Banks S.J., Bayram E., Shan G., LaBelle D.R., Bluett B. (2019). Non-motor predictors of freezing of gait in Parkinson’s disease. Gait Posture.

[50] Delgado-Alvarado M., Marano M., Santurtún A., Urtiaga-Gallano A., Tordesillas-Gutierrez D., Infante J. (2020). Nonpharmacological, nonsurgical treatments for freezing of gait in Parkinson’s disease: A systematic review. Mov. Disord. Off. J. Mov. Disord. Soc..

[51] Droby A., Pelosin E., Putzolu M., Bommarito G., Marchese R., Mazzella L., Avanzino L., Inglese M. (2021). A Multimodal Imaging Approach Demonstrates Reduced Midbrain Functional Network Connectivity Is Associated With Freezing of Gait in Parkinson’s Disease. Front. Neurol..

[52] Grabli D., Karachi C., Welter M.-L., Lau B., Hirsch E.C., Vidailhet M., François C. (2012). Normal and pathological gait: What we learn from Parkinson’s disease. J. Neurol. Neurosurg. Psychiatry.

[53] Wang M., Jiang S., Yuan Y., Zhang L., Ding J., Wang J., Zhang J., Zhang K., Wang J. (2016). Alterations of functional and structural connectivity of freezing of gait in Parkinson’s disease. J. Neurol..

[54] Lv L., Zhang H., Tan X., Long Z., Qin L., Bai R., Xiao Q., Wu Z., Hu S., Tan C. (2022). Associated factors and abnormal dorsal raphe nucleus connectivity patterns of freezing of gait in Parkinson’s disease. J. Neurol..

[55] Briand L.A., Gritton H., Howe W.M., Young D.A., Sarter M. (2007). Modulators in concert for cognition: Modulator interactions in the prefrontal cortex. Prog. Neurobiol..

[56] Canu E., Agosta F., Sarasso E., Volontè M.A., Basaia S., Stojkovic T., Stefanova E., Comi G., Falini A., Kostic V.S. (2015). Brain structural and functional connectivity in P arkinson’s disease with freezing of gait. Hum. Brain Mapp..

[57] Lenka A., Naduthota R.M., Jha M., Panda R., Prajapati A., Jhunjhunwala K., Saini J., Yadav R., Bharath R.D., Pal P.K. (2016). Freezing of gait in Parkinson’s disease is associated with altered functional brain connectivity. Parkinsonism Relat. Disord..

[58] Guo M., Ren Y., Yu H., Yang H., Cao C., Li Y., Fan G. (2020). Alterations in Degree Centrality and Functional Connectivity in Parkinson’s Disease Patients with Freezing of Gait: A Resting-State Functional Magnetic Resonance Imaging Study. Front. Neurosci..

[59] Wang S., Cai H., Cao Z., Li C., Wu T., Xu F., Qian Y., Chen X., Yu Y. (2021). More Than Just Static: Dynamic Functional Connectivity Changes of the Thalamic Nuclei to Cortex in Parkinson’s Disease with Freezing of Gait. Front. Neurol..

[60] Bharti K., Suppa A., Pietracupa S., Upadhyay N., Giannì C., Leodori G., Di Biasio F., Modugno N., Petsas N., Grillea G. (2019). Abnormal Cerebellar Connectivity Patterns in Patients with Parkinson’s Disease and Freezing of Gait. The Cerebellum.

[61] Quek D.Y.L., Taylor N., Gilat M., Lewis S.J.G., Ehgoetz Martens K.A. (2024). Effect of dopamine on limbic network connectivity at rest in Parkinson’s disease patients with freezing of gait. Transl. Neurosci..

[62] Lench D.H., Embry A., Hydar A., Hanlon C.A., Revuelta G. (2020). Increased on-state cortico-mesencephalic functional connectivity in Parkinson disease with freezing of gait. Park. Relat. Disord..

[63] Reijnders J.S.A.M., Ehrt U., Lousberg R., Aarsland D., Leentjens A.F.G. (2009). The association between motor subtypes and psychopathology in Parkinson’s disease. Park. Relat. Disord..

[64] Moustafa A.A., Bell P., Eissa A.M., Hewedi D.H. (2013). The effects of clinical motor variables and medication dosage on working memory in Parkinson’s disease. Brain Cogn..

[65] Sridharan D., Levitin D.J., Menon V. (2008). A critical role for the right fronto-insular cortex in switching between central-executive and default-mode networks. Proc. Natl. Acad. Sci. USA.

[66] Wang J., Shen Y., Peng J., Wang A., Wu X., Chen X., Liu J., Wei M., Zou D., Han Y. (2021). Different functional connectivity modes of the right fronto-insular cortex in akinetic-rigid and tremor-dominant Parkinson’s disease. Neurol. Sci..

[67] van Eimeren T., Monchi O., Ballanger B., Strafella A.P. (2009). Dysfunction of the default mode network in Parkinson disease: A functional magnetic resonance imaging study. Arch. Neurol..

[68] Hou Y., Luo C., Yang J., Ou R., Liu W., Song W., Gong Q., Shang H. (2017). Default-mode network connectivity in cognitively unimpaired drug-naïve patients with rigidity-dominant Parkinson’s disease. J. Neurol..

[69] Hu X., Jiang Y., Jiang X., Zhang J., Liang M., Li J., Zhang Y., Yao D., Luo C., Wang J. (2017). Altered Functional Connectivity Density in Subtypes of Parkinson’s Disease. Front. Hum. Neurosci..

[70] Schuepbach W.M.M., Rau J., Knudsen K., Volkmann J., Krack P., Timmermann L., Hälbig T.D., Hesekamp H., Navarro S.M., Meier N. (2013). Neurostimulation for Parkinson’s disease with early motor complications. N. Engl. J. Med..

[71] Bai Y., Diao Y., Gan L., Zhuo Z., Yin Z., Hu T., Cheng D., Xie H., Wu D., Fan H. (2022). Deep Brain Stimulation Modulates Multiple Abnormal Resting-State Network Connectivity in Patients With Parkinson’s Disease. Front. Aging Neurosci..

[72] Horn A., Reich M., Vorwerk J., Li N., Wenzel G., Fang Q., Schmitz-Hübsch T., Nickl R., Kupsch A., Volkmann J. (2017). Connectivity Predicts deep brain stimulation outcome in P arkinson disease. Ann. Neurol..

[73] Caire F., Ranoux D., Guehl D., Burbaud P., Cuny E. (2013). A systematic review of studies on anatomical position of electrode contacts used for chronic subthalamic stimulation in Parkinson’s disease. Acta Neurochir..

[74] Koirala N., Fleischer V., Granert O., Deuschl G., Muthuraman M., Groppa S. (2016). Network effects and pathways in Deep brain stimulation in Parkinson’s disease. Proceedings of the 2016 38th Annual International Conference of the IEEE Engineering in Medicine and Biology Society (EMBC).

[75] Accolla E.A., Herrojo Ruiz M., Horn A., Schneider G.-H., Schmitz-Hübsch T., Draganski B., Kühn A.A. (2016). Brain networks modulated by subthalamic nucleus deep brain stimulation. Brain.

[76] Hollunder B., Ostrem J.L., Sahin I.A., Rajamani N., Oxenford S., Butenko K., Neudorfer C., Reinhardt P., Zvarova P., Polosan M. (2024). Mapping dysfunctional circuits in the frontal cortex using deep brain stimulation. Nat. Neurosci..

[77] Calvano A., Kleinholdermann U., Heun A.-S., Bopp M.H.A., Nimsky C., Timmermann L., Pedrosa D.J. (2024). Structural connectivity of low-frequency subthalamic stimulation for improving stride length in Parkinson’s disease. NeuroImage Clin..

[78] Chen H., Sha Z., Ma H., He Y., Feng T. (2018). Effective network of deep brain stimulation of subthalamic nucleus with bimodal positron emission tomography/functional magnetic resonance imaging in Parkinson’s disease. CNS Neurosci. Ther..

[79] Shen L., Jiang C., Hubbard C.S., Ren J., He C., Wang D., Dahmani L., Guo Y., Liu Y., Xu S. (2020). Subthalamic Nucleus Deep Brain Stimulation Modulates 2 Distinct Neurocircuits. Ann. Neurol..

[80] Rajamani N., Friedrich H., Butenko K., Dembek T., Lange F., Navrátil P., Zvarova P., Hollunder B., De Bie R.M.A., Odekerken V.J.J. (2024). Deep brain stimulation of symptom-specific networks in Parkinson’s disease. Nat. Commun..

[81] Strelow J.N., Baldermann J.C., Dembek T.A., Jergas H., Petry-Schmelzer J.N., Schott F., Dafsari H.S., Moll C.K.E., Hamel W., Gulberti A. (2022). Structural Connectivity of Subthalamic Nucleus Stimulation for Improving Freezing of Gait. J. Park. Dis..

[82] DiMarzio M., Madhavan R., Joel S., Hancu I., Fiveland E., Prusik J., Gillogly M., Rashid T., MacDonell J., Ashe J. (2020). Use of Functional Magnetic Resonance Imaging to Assess How Motor Phenotypes of Parkinson’s Disease Respond to Deep Brain Stimulation. Neuromodul. Technol. Neural Interface.

[83] Odekerken V.J.J., van Laar T., Staal M.J., Mosch A., Hoffmann C.F.E., Nijssen P.C.G., Beute G.N., van Vugt J.P.P., Lenders M.W.P.M., Contarino M.F. (2013). Subthalamic nucleus versus globus pallidus bilateral deep brain stimulation for advanced Parkinson’s disease (NSTAPS study): A randomised controlled trial. Lancet Neurol..

[84] Odekerken V.J.J., Boel J.A., Schmand B.A., de Haan R.J., Figee M., van den Munckhof P., Schuurman P.R., de Bie R.M.A., NSTAPS study Group (2016). GPi vs STN deep brain stimulation for Parkinson disease: Three-year follow-up. Neurology.

[85] DiRisio A.C., Avecillas-Chasin J.M., Platt S., Jimenez-Shahed J., Figee M., Mayberg H.S., Choi K.S., Kopell B.H. (2023). White matter connectivity of subthalamic nucleus and globus pallidus interna targets for deep brain stimulation. J. Neurosurg..

[86] Li Z., Lai Y., Li J., He N., Li D., Yan F., Zhang Y., Zhang C., Sun B., Wei H. (2023). BOLD frequency–dependent alterations in resting-state functional connectivity by pallidal deep brain stimulation in patients with Parkinson’s disease. J. Neurosurg..

[87] Fox M.D. (2018). Mapping Symptoms to Brain Networks with the Human Connectome. N. Engl. J. Med..

[88] Hariz M., Blomstedt P. (2022). Deep brain stimulation for Parkinson’s disease. J. Intern. Med..

